# Antenatal Intravenous Iron Therapy in Anemic Pregnancies: Associations with Obstetric Patient Blood Management and Neonatal Outcomes

**DOI:** 10.3390/nu18193169

**Published:** 2026-09-26

**Authors:** Eleonora Romani, Francesca Parisi, Sara Zullino, Chiara Lubrano, Silvia Balestri, Clara Massari, Salvatore Barresi, Massimo Micaglio, Felice Petraglia, Irene Cetin, Federico Mecacci

**Affiliations:** 1 Obstetrics and Gynaecology Unit, Department of Experimental and Clinical Biomedical Sciences, University of Florence, 50134 Florence, Italy; eleonora.romani@unifi.it (E.R.);; 2 Complex Obstetrics Unit, Fondazione IRCCS Cà Granda, Ospedale Maggiore Policlinico, 20122 Milan, Italy; 3 Department of Biomedical and Clinical Sciences, University of Milan, 20157 Milan, Italy; 4 High Risk Pregnancy Unit, Department for Women and Health, Careggi University Hospital, 50134 Florence, Italy; 5 Nutritional Sciences-Doctoral Programme, Department of Veterinary Medicine and Animal Sciences (DIVAS), University of Milan, 26900 Lodi, Italy; 6 Department of Anesthesia and Intensive Care, Careggi University Hospital, 50134 Florence, Italy; 7 Department of Clinical and Community Sciences, University of Milan, 20122 Milan, Italy

**Keywords:** pregnancy, anemia, iron deficiency, infant, blood transfusion

## Abstract

**Background:** Anemia in pregnancy is associated with increased maternal and neonatal morbidity, most commonly due to iron deficiency. The effect of antenatal IV iron therapy on obstetric patient blood management (PBM) and neonatal outcomes remains uncertain. **Objective:** To evaluate whether antenatal IV iron therapy in pregnancy is associated with improved obstetric PBM, specifically reduced RBC transfusion and postpartum IV iron use, and improved neonatal outcomes. **Methods:** In this multicenter retrospective study, 851 pregnant women with IDA were included (490 received antenatal IV iron; 361 controls). Multivariable logistic regression adjusted for clinically relevant confounders assessed maternal and neonatal outcomes. A propensity score-adjusted sensitivity analysis addressed baseline differences. **Results:** The median gestational age at infusion was 35 weeks (range 30–37). The treated group had a higher obstetric risk profile, lower baseline hemoglobin at 9.2 (range 5.5–12.7) vs. 9.8 (range 7.9–12) g/dL, and more frequent oral iron use (30–100 mg/day; 66.1% vs. 53.7%; *p* < 0.001). Gestational age at delivery, estimated blood loss, and postpartum hemorrhage were similar between groups. After adjustment, antenatal IV iron therapy was associated with 56% and 72% lower odds of RBC transfusion and postpartum IV iron use, respectively; the reduced odds of blood transfusion remained significant after propensity score adjustment (aOR 0.42, 95%CI 0.16–0.99). A significant interaction with baseline hemoglobin was observed, with benefit mainly in women with a lower baseline. Neonatal outcomes were similar between groups, although NICU/high-dependency admission and phototherapy for jaundice were less frequent among neonates of treated women. **Conclusions:** Antenatal IV iron therapy was associated with reduced transfusion exposure and lower postpartum IV iron requirements, despite lower baseline Hb values. These findings support antenatal hematologic optimization in obstetric PBM and highlight the need for further studies to clarify potential neonatal benefits.

## 1. Introduction

Anemia is a major global health concern, affecting 32 million pregnant women worldwide [1], with iron deficiency (ID) representing its leading cause [2]. The International Federation of Gynecology and Obstetrics (FIGO) recently defined anemia in pregnancy as hemoglobin (Hb) < 11.0 g/dL across all trimesters and the postpartum period [3], introducing a unified threshold to harmonize previously heterogeneous international guidelines [4,5] and improve consistency in diagnosis and management.

Untreated maternal iron deficiency anemia (IDA) has been associated with adverse obstetric and neonatal outcomes, including preeclampsia [6,7], preterm birth [8], postpartum hemorrhage (PPH) [9,10], peripartum transfusion [11,12], fetal growth restriction (FGR), low birth weight, and neonatal intensive care unit (NICU) admission [13,14,15,16,17]. These associations have reinforced the importance of the early detection and correction of maternal anemia.

The optimization of the maternal Hb concentration before delivery has become a central component of obstetric patient blood management (PBM) [18,19], an approach aimed at enhancing maternal hematologic reserve at delivery and minimizing transfusion requirements and corrective postpartum interventions. In this context, oral iron supplementation remains the first-line therapy for IDA in pregnancy. However, gastrointestinal intolerance and insufficient time to achieve adequate Hb correction, particularly in late gestation, may reduce its effectiveness [20]. When rapid iron repletion is required or oral therapy is insufficient and/or not tolerated, intravenous (IV) iron formulations provide a more efficient alternative with demonstrated hematologic efficacy [21,22,23]. Despite its hematologic efficacy, evidence that antenatal IV iron therapy improves maternal and neonatal clinical outcomes remains limited [24,25,26,27]. Moreover, IV iron is frequently administered to women with moderate-to-severe anemia (Hb < 9.9 g/dL) [3,5] or higher obstetric risk profiles, introducing potential confounding by indication in observational studies and complicating the interpretation of outcome data. Clarifying the real-world impact of antenatal IV iron therapy on both obstetric PBM metrics and neonatal outcomes is therefore of substantial clinical and health-policy relevance.

The aim of the present study was to evaluate the association between antenatal IV iron therapy for maternal IDA and maternal PBM and neonatal outcomes.

The primary outcome was the requirement for red blood cell (RBC) transfusion. Secondary maternal outcomes included PPH and the need for postpartum IV iron therapy.

The secondary neonatal outcomes included the five-minute Apgar score, umbilical artery pH, NICU or high-dependency care admission, perinatal death, and neonatal jaundice requiring treatment.

## 2. Materials and Methods

### 2.1. Study Design

This multicenter retrospective cohort study was carried out at two tertiary referral centers, Careggi University Hospital (Florence, Italy) and Vittore Buzzi Children’s Hospital (Milan, Italy), between 2019 and 2025. At the time of hospital admission, all participants provided written informed consent for IV iron administration, including authorization for the secondary use of routinely collected clinical data for research purposes, independently of the present study. Clinical data were retrospectively extracted from medical records by authorized physicians at each participating center and entered directly into a coded study database without direct patient identifiers. The absence of IRB review was based on the internal policies of both participating institutions, under which approval was not required for this type of retrospective observational study.

### 2.2. Study Population

Women undergoing IV iron therapy with Ferric Carboxymaltose (FCM) during the second or third trimester of pregnancy were eligible for enrollment (treated group). Indications for IV iron administration included one or more of the following: (i) moderate to severe IDA, defined by a Hb level ≤ 9.9 g/dL in combination with serum ferritin concentrations < 30 ng/mL; (ii) intolerance to and/or an inadequate response to prior oral iron therapy; (iii) a clinical need for rapid iron repletion; (iv) mild anemia (Hb < 11 g/dL) in the third trimester with coexisting maternal risk factors for PPH (i.e., previous PPH, placenta accreta spectrum (PAS)); (v) the presence of anemia-related symptoms, such as fatigue, dyspnea, or palpitations. The exclusion criteria comprised any of the following: (i) anemia attributable to causes other than ID (e.g., thalassemia, hemolytic anemia, or anemia related to chronic disease); (ii) contraindications to IV iron therapy (e.g., active infection, severe hepatic disease, or renal failure); (iii) documented hypersensitivity to FCM or its excipients; (iv) anemia diagnosed in the first trimester of pregnancy; (v) incomplete outcome data.

Untreated controls were identified from women diagnosed with IDA delivering at the participating centers during the same study period who did not receive IV iron during pregnancy and fulfilled the study eligibility criteria. Adjustment strategies were used to account for baseline differences in anemia severity and clinical profile. No predefined matching ratio was applied. The final sample size reflected the number of eligible untreated women available during the study period and the real-world distribution of IV iron use in tertiary referral centers.

### 2.3. Treatment Protocol

Women in the treated group received FCM administered intravenously at a dose of 500 mg diluted in 250 mL of normal saline and infused over 15 min. Individual doses did not exceed 20 mg/kg body weight and the maximum cumulative dose was limited to 1000 mg per week, generally delivered in one or two infusions. Oral iron supplementation was suspended during the week following IV administration. No specific dietary recommendations or additional nutritional interventions were prescribed.

### 2.4. Maternal and Neonatal Data Collection

Baseline maternal demographic characteristics, chronic comorbidities, pregnancy complications (i.e., gestational diabetes (GDM), hypertensive disorders of pregnancy (HDPs), placenta accreta spectrum (PAS), infectious disease in pregnancy), use of aspirin and low-molecular-weight heparin, and oral iron supplements in pregnancy were documented for both groups. Baseline Hb levels were measured immediately before IV iron administration in the treated group and at a corresponding gestational age (±two weeks) in the control group. Delivery data (i.e., mode of delivery, gestational age at delivery, labor induction, use of epidural analgesia, PPH, Bakri balloon use, need for RBC transfusion, postpartum IV and oral iron therapy) were extracted from medical records at the time of postpartum hospital discharge.

Neonatal outcomes including five-minute Apgar score, umbilical artery pH, NICU or high-dependency care admission, perinatal death, and neonatal jaundice requiring treatment were recorded.

### 2.5. Statistical Analysis

Given the retrospective and observational design of the study, the sample size was determined by the number of eligible women treated at the participating centers during the study period, representing a convenience sample. No a priori sample size calculation was performed. Descriptive statistics were used to summarize the patient characteristics and outcome data of the two study groups. The distribution of continuous variables was assessed using the Shapiro–Wilk test and visual inspection of Q–Q plots. As the principal continuous variables showed departures from normality, continuous variables are presented as medians and ranges and were compared between groups using the Mann–Whitney U test. Categorical variables are presented as absolute and relative frequencies and were compared using the chi-square test or Fisher’s exact test, as appropriate.

First, multivariable binary logistic regression models were used to identify maternal baseline characteristics, chronic comorbidities, and pregnancy-related factors associated with IV iron treatment, with treatment group specified as the dependent variable.

Second, multivariable binary logistic regression models were used to evaluate the association between IV iron treatment (independent variable) and the primary outcome, namely the need for blood transfusion (dependent variable). Models were adjusted for clinically relevant confounders, including maternal age, pregestational body mass index (BMI), parity, mode of conception, multiple pregnancy, oral iron therapy, baseline Hb concentrations, gestational age at delivery, and mode of delivery.

Secondary binary outcomes (Bakri balloon use, need for postpartum IV iron therapy, PPH, cesarean delivery, NICU admission or high-dependency care admission, neonatal jaundice) were analyzed using similar logistic regression models, while continuous outcomes (birth weight) were evaluated using multivariable generalized linear models. For the primary outcome, effect modification by baseline Hb level was assessed by including an interaction term between baseline Hb and treatment group in the regression model. To facilitate the clinical interpretation of the interaction, additional exploratory stratified analyses were performed according to baseline Hb categories defined by the 25th, 50th, and 75th percentiles of the observed baseline Hb distribution. Within each Hb category, the association between IV iron treatment and the risk of blood transfusion was evaluated using multivariable logistic regression models adjusted for the same set of prespecified confounders as in the primary analysis. The results are reported as corresponding adjusted odds ratios (aORs) or β-coefficients with 95% confidence intervals (CIs). Because only 36 RBC transfusion events occurred, the primary model was considered at risk of overfitting; the interaction and stratified analyses were therefore interpreted as exploratory.

As a sensitivity analysis, propensity score overlap weighting was used to further address differences in baseline characteristics between women who received IV iron treatment and controls.

Treatment group assignment was modeled as the dependent variable and baseline covariates—including maternal age, pregestational BMI, gestational weight gain, prior cesarean delivery, ethnicity, mode of conception, multiple pregnancy, prior bariatric surgery, benign gynecological conditions (i.e., endometriosis, uterine fibroids, adenomyosis), hematologic disorders (e.g., Von Willebrand disease, thrombocytopenia, hemophilia), pregnancy complications (composite variable including GDM, HDP, FGR, and intrahepatic cholestasis of pregnancy), and baseline Hb level—were included as predictors. Women with missing data for any propensity score covariate were excluded from this analysis. Overlap weights were calculated as 1 minus the propensity score for treated women and as the propensity score for controls. The propensity score distributions were examined graphically, and empirical common support was assessed using absolute standardized mean differences, with values below 0.10 considered indicative of adequate balance. The association between IV iron treatment and need for blood transfusion was subsequently estimated using an overlap-weighted generalized linear model with a binomial distribution, logit link, and robust covariance estimator. Statistical analyses were performed using SPSS Statistics software version 29.0 (IBM Corp, Chicago, IL, USA). *p*-values < 0.05 were considered statistically significant.

## 3. Results

During the study period, the two centers recorded 43,840 deliveries overall. A total of 851 women were included during the study period, of whom 490 received antenatal IV iron therapy and 361 served as controls. No serious maternal adverse events were recorded. Table 1 shows the maternal and pregnancy characteristics with comparisons between the two study groups. In detail, treated women were older and more frequently diagnosed with chronic comorbidities such as previous bariatric surgery, benign gynecological disorders, and hematologic disorders. Furthermore, pregnancies of the treated group were more frequently multiple, conceived through assisted reproductive technology (ART), complicated by GDM and PAS disorders, and under aspirin or heparin treatment. Conversely, the control group showed higher rates of inadequate prenatal care, defined as late initiation of antenatal follow-up (>28 weeks) and/or less than four prenatal visits, and/or non-continuous antenatal care, compared to the treated group. The median gestational age at infusion was 35 weeks (IQR 30–37). The baseline Hb concentrations measured at the time of IV iron therapy were significantly lower in the treated than control group, with a four-fold higher rate of moderate to severe anemia in the same group.

Figure 1 summarizes the results of the multivariable logistic regression analysis evaluating the association between maternal and pregnancy-related characteristics and IV iron treatment (dependent variable). Multiple pregnancy, prior bariatric surgery, benign gynecological conditions, and PAS disorders were significantly associated with higher odds of receiving IV iron treatment. In contrast, higher baseline Hb levels and greater gestational weight gain were independently associated with lower odds of IV iron treatment during pregnancy.

The delivery outcome data are summarized in Table 2. Gestational age at delivery, blood loss, rates of PPH, and blood transfusion did not differ between the treated and control groups. Conversely, induction of labor, use of epidural analgesia, and vaginal delivery occurred less frequently among treated women, with a corresponding higher rate of cesarean delivery in the same group compared to controls. Bakri balloon placement was more frequent in the treated group, while postpartum Hb levels were significantly higher among treated women. Accordingly, the use of postpartum IV iron therapy was lower in the treated group, as was the use of oral iron supplementation.

Neonatal outcomes were largely comparable between groups, except for NICU or high-dependency care admission, which occurred less frequently in neonates born to treated women compared with controls (19.0% vs. 24.4%), reaching borderline statistical significance (*p* = 0.05).

Table 3 presents the results of multivariable binary logistic regression and generalized linear models examining the associations between treatment group, baseline Hb concentrations, and delivery outcomes. After adjustment for the prespecified confounders, significant differences were observed between treated and control women for several delivery outcomes. For the primary outcome, women who received antenatal IV iron therapy had 58% lower adjusted odds of requiring RBC transfusion compared with controls. Among the secondary maternal outcomes, IV iron therapy was associated with 72% lower adjusted odds of postpartum IV iron administration. No statistically significant associations were observed between treatment group and PPH, birth weight, or cesarean delivery.

Regarding neonatal outcomes, neonates born to treated mothers had a significantly lower risk of developing jaundice, as well as a reduced likelihood of admission to the NICU or high-dependency care compared with controls. Baseline Hb concentrations were significantly associated with reduced odds of requiring postpartum IV iron therapy, with no other significant associations being detected.

The inclusion of the interaction term between treatment group and baseline Hb concentration in the primary model indicated a significant effect modification by baseline Hb levels (*p* < 0.01). To further explore the observed interaction between baseline Hb concentration and treatment group, additional stratified analyses were performed according to baseline Hb categories defined by the 25th, 50th, and 75th percentiles of the baseline Hb distribution (8.9, 9.5, and 9.9 g/dL, respectively). In these analyses, antenatal IV iron therapy was significantly associated with a lower likelihood of RBC transfusion only among women with baseline Hb levels below the 25th percentile (<8.9 g/dL). In this subgroup, IV iron therapy was associated with a markedly reduced risk of transfusion (aOR 0.09, 95% CI 0.02–0.42; *p* < 0.01). No statistically significant associations were observed in the higher baseline Hb strata.

The propensity score sensitivity analysis included 799 women with complete covariate data, comprising 481 treated and 318 control women. The propensity score distributions showed substantial overlap between groups. Overlap weighting achieved excellent balance across all prespecified baseline covariates. After weighting, the absolute standardized mean differences were below 0.10 for every covariate and rounded to 0.00 at the reported precision. In the overlap-weighted outcome model, IV iron treatment was associated with lower odds of blood transfusion, suggesting that baseline differences between treated and control groups did not materially influence the observed associations.

## 4. Discussion

### 4.1. Main Findings

After adjustment for clinically relevant confounders, women receiving IV iron had substantially lower odds of requiring RBC transfusion and postpartum IV iron therapy compared with untreated controls. These findings suggest that antenatal IV iron therapy for maternal IDA may contribute to reducing postpartum transfusion exposure and the need for additional iron therapy after delivery. Importantly, the association between antenatal IV iron therapy and transfusion risk appeared to vary according to baseline anemia severity. The interaction analysis demonstrated significant effect modification by baseline Hb levels, and stratified analyses indicated that the protective association of IV iron therapy was primarily observed among women with the lowest baseline Hb concentrations. In particular, women with baseline Hb levels below the 25th percentile (<8.9 g/dL) had markedly lower adjusted odds of transfusion when treated antenatally with IV iron. In contrast, no significant association was observed among women with higher baseline Hb values. These findings suggest that the potential benefit of antenatal IV iron therapy for transfusion risk may be greatest among women with more severe anemia. Nevertheless, the number of transfusion events within individual strata was limited, resulting in potential unstable effect estimates.

IDA in pregnancy has consistently been associated with an increased risk of adverse obstetric outcomes, including PPH, particularly due to uterine atony [9]. Proposed mechanisms include adaptive placental changes in response to reduced maternal oxygen-carrying capacity, such as enhanced trophoblastic invasion [28,29] and increased production of vasodilatory mediators including nitric oxide [30], which may exert a paracrine inhibitory effect on myometrial contractility, with consequent impaired uterine tone during the third stage of labor and increased bleeding from the placental site [31,32,33].

In our cohort, women who received antenatal IV iron therapy, despite having a higher baseline obstetric risk profile—characterized by increased rates of multiple pregnancy, a higher number of cesarean deliveries, PAS, ART conception, and maternal comorbidities—did not experience a higher rate of PPH compared with the controls, while showing a 60% reduction in the odds of RBC transfusion and a 72% reduction in postpartum IV iron therapy. This apparent dissociation between unchanged hemorrhage incidence and reduced transfusion exposure suggests that antenatal IV iron does not prevent hemorrhage per se, but rather improves maternal hematologic reserve at delivery, thereby mitigating the clinical consequences of peripartum blood loss and reducing the need for therapeutic interventions in the postpartum period. This interpretation is consistent with current evidence. A recent Cochrane review [24] showed that IV iron improves Hb levels, but results in little to no difference in PPH compared with oral iron. Similarly, recent meta-analyses reported superior hematologic efficacy of IV iron without consistent reductions in individual hemorrhage outcomes [22,34]. Recent real-world data, consistent with our findings, showed that the administration of IV iron more than 10 days before delivery is associated with higher Hb levels and reduced transfusion requirements, underscoring the clinical relevance of pre-delivery hematologic optimization [35]. These observations align with the principles of PBM, which emphasize the antenatal identification and treatment of IDA to minimize transfusion exposure and its associated risks, with potential implications for healthcare resource utilization and costs [19]. Moreover, the available evidence also supports a favorable safety profile of IV iron therapy during pregnancy [36,37].

Maternal anemia has been associated with increased neonatal morbidity, including higher rates of NICU admission [38]. However, evidence regarding the impact of maternal IV iron therapy on neonatal clinical outcomes remains limited and inconsistent.

Recent observational and population-based cohort studies have reported that the treatment of maternal anemia is associated with a reduced risk of NICU admission [39], while the hematologic response to iron therapy has been associated with reduced risks of preterm birth and preeclampsia [25]—conditions closely related to neonatal morbidity—suggesting that hematologic optimization may influence neonatal risk. In contrast, randomized trials and meta-analyses [24,40,41] comparing IV and oral iron consistently demonstrate improvements in maternal hematologic indices but have not shown clear reductions in major neonatal outcomes such as birth weight, gestational age, or composite neonatal morbidity.

In our cohort, antenatal IV iron therapy was not associated with differences in gestational age at delivery, birth weight, perinatal mortality, five-minute Apgar score, or umbilical arterial pH, indicating no measurable impact on fetal growth or neonatal adaptations. However, IV iron therapy was independently associated with a lower likelihood of NICU or high-dependency care admission and reduced neonatal jaundice requiring treatment, even after adjustment for baseline maternal and obstetric characteristics. Given the observational design, these findings should be interpreted cautiously and may represent chance findings. Prospective studies specifically powered for neonatal endpoints are warranted.

### 4.2. Strengths and Limitations

This study has several limitations. First, its retrospective observational design precludes causal inference and is inherently subject to residual confounding despite adjustment for multiple clinically relevant covariates in multivariable models. In particular, treatment allocation was not randomized and was driven by clinical indication, raising the possibility of confounding by indication. To mitigate this bias, multivariable adjustment was applied and a propensity score sensitivity analysis was performed, which yielded consistent results for the primary outcome.

Second, the composition of the control group represents an additional limitation. Untreated controls were identified among women delivering during the same study period who did not receive IV iron therapy, regardless of baseline anemia severity. Because IV iron was preferentially administered to women with more severe anemia or inadequate responses to oral iron, the control group included women with milder anemia at baseline. This imbalance may have attenuated between-group differences despite statistical adjustment, and residual confounding cannot be excluded.

Third, the number of RBC transfusion events was limited relative to the number of estimated regression parameters, increasing the possibility of model overfitting and unstable effect estimates. This concern is particularly relevant to the treatment-by-baseline-hemoglobin interaction and the hemoglobin-stratified analyses, in which few events occurred within individual strata. These analyses should therefore be considered exploratory and interpreted cautiously. Although propensity score overlap weighting produced excellent balance in the measured baseline covariates, it cannot account for unmeasured or inaccurately measured confounding. In addition, the propensity score analysis was restricted to participants with complete covariate data. Finally, as with most retrospective studies, the data completeness and accuracy depended on the quality of medical records, and some potentially relevant variables—such as adherence to prior oral iron therapy or the exact timing of anemia onset—may not have been fully captured.

Despite these limitations, this study also has important strengths. It includes a relatively large multicenter cohort, supporting the clinical relevance of the findings in tertiary-care obstetric settings, although generalizability to other populations may be limited. Baseline Hb levels were measured immediately before IV iron administration in the treated group and at a corresponding gestational age in the control group, improving comparability between groups. In addition, the analytical strategy combined conventional multivariable modeling, formal assessment of interactions by baseline anemia severity, and propensity score-adjusted sensitivity analysis to address treatment-selection bias. Finally, by simultaneously evaluating maternal PBM outcomes and neonatal endpoints, this study provides a clinically integrated assessment of the potential impact of antenatal IV iron therapy in routine obstetric practice.

## 5. Conclusions

This multicenter observational study showed that antenatal IV iron therapy for IDA treatment was independently associated with reduced RBC transfusion and postpartum IV iron, supporting its potential role in antenatal hematologic optimization within obstetric care.

Although neonatal outcomes were largely comparable between groups, the observed association with reduced NICU admission and neonatal jaundice suggests that the correction of maternal ID may have implications extending beyond maternal hematologic parameters. While causality cannot be established in this observational setting, these findings warrant further prospective investigation specifically focused on neonatal endpoints.

Taken together, our results support a potential role of antenatal IV iron therapy as an effective hematologic optimization strategy in pregnant women with IDA, and, in light of existing safety evidence, suggest an association with reduced transfusion exposure and potentially lower healthcare resource utilization.

## Figures and Tables

**Figure 1:**
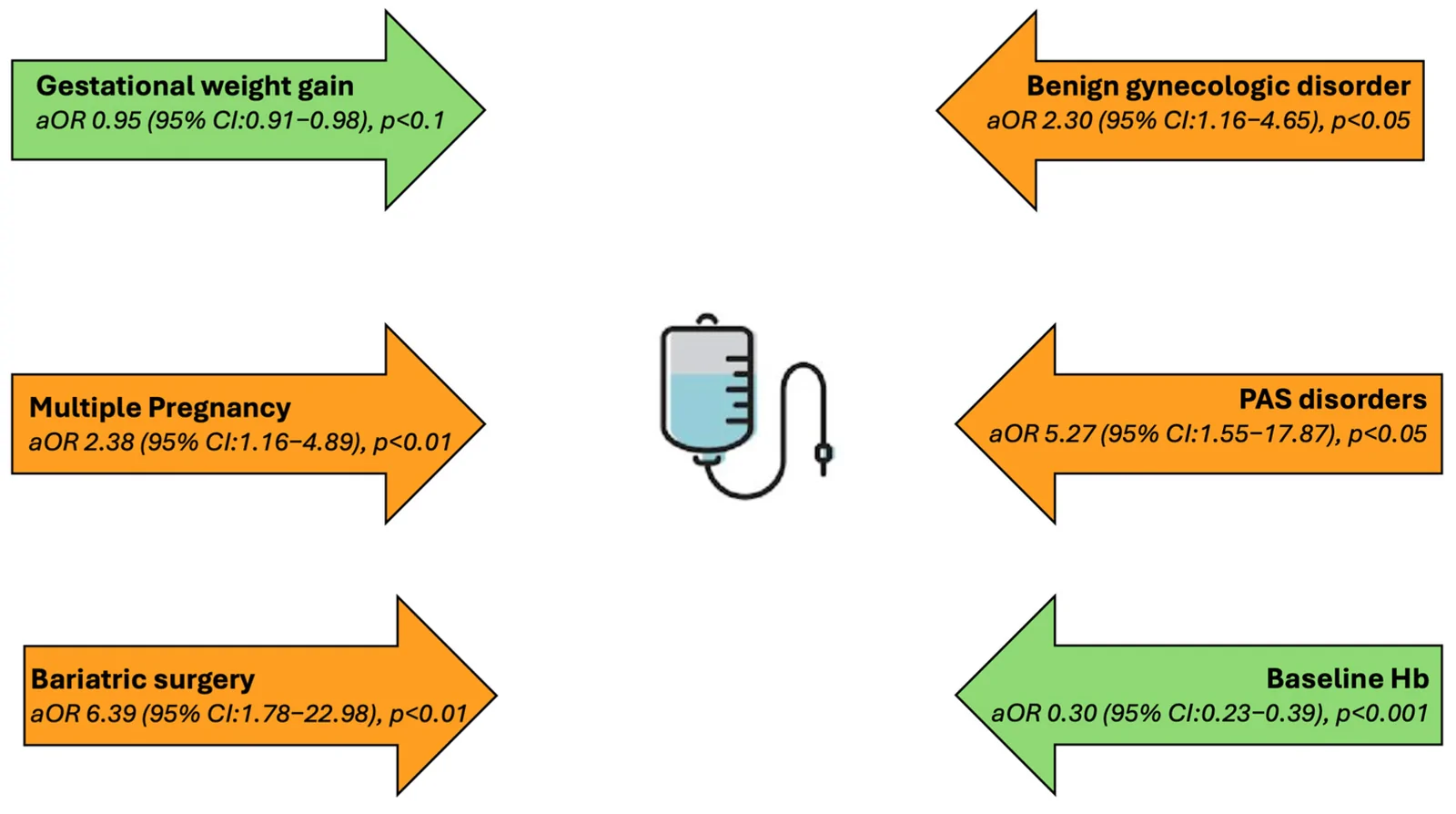
Graphical representation of logistic regression models defining risk factors for intravenous iron therapy in pregnancy. PAS: placenta accreta spectrum. Green arrows highlight characteristics associated with a reduced risk of intravenous iron therapy. Orange arrows highlight characteristics associated with an increased risk of intravenous iron therapy. Logistic regression models included—besides the shown variables—adjustment for maternal age, pregestational BMI, conception mode, parity, and previous cesarean delivery.

**Table 1:** Maternal demographic and clinical data with comparisons between the study groups.

Characteristic	Treated Group (N = 490)	Control Group (N = 361)	*p*-Value
Age, years, median (range)	35 (15–52)	33 (18–50)	<0.01
Pregestational BMI, kg/m^2^, median (range)	22.4 (13.0–51.9)	22.0 (15.0–40.0)	0.72
Gestational weight gain, kg, median (range)	10.0 (0.0–36.0)	12.0 (−3.0–33.0)	<0.01
Smoking habit, n (%)	12 (2.7)	13 (3.3)	0.56
Nulliparous, n (%)	231 (47.1)	164 (45.4)	0.62
Previous cesarean delivery, n (%)	101 (20.6)	67 (18.6)	0.46
Caucasian ethnicity, n (%)	369 (75.3)	264 (73.1)	0.29
Multiple pregnancy, n (%)	39 (8)	12 (3.3)	<0.01
Previous bariatric surgery, n (%)	24 (4.9)	3 (0.8)	<0.001
Benign uterine gynecological conditions, n (%)	51 (10.4)	13 (3.6)	<0.001
GI disorders, n (%)	28 (5.7)	13 (3.6)	0.15
Chronic hypertension, n (%)	7 (1.4)	9 (2.5)	0.26
Hematologic disorders, n (%)	11 (2.2)	1 (0.3)	<0.05
Respiratory disorders, n (%)	23 (4.7)	14 (3.9)	0.56
Pregestational DM, n (%)	18 (3.7)	12 (3.3)	0.78
Hypothyroidism, n (%)	21 (4.3)	9 (2.5)	0.16
Psychiatric disorders, n (%)	4 (0.8)	6 (1.7)	0.25
ART conception, n (%)	69 (14.1)	29 (8.0)	<0.01
GDM, n (%)	105 (21.4)	58 (16.1)	<0.05
HDP, n (%)	22 (4.5)	16 (4.4)	0.96
PAS, n (%)	30 (6.1)	3 (0.8)	<0.001
Inadequate prenatal care, n (%)	5 (1)	22 (6.1)	<0.001
Infectious disease in pregnancy, n (%)	9 (1.8)	15 (4.2)	<0.05
Aspirin use in pregnancy, n (%)	127 (25.9)	72 (19.9)	<0.05
Low-molecular-weight heparin use in pregnancy, n (%)	104 (21.2)	53 (14.7)	<0.05
Baseline Hb, g/dL, median (range)	9.2 (5.5–12.7)	9.8 (7.9–12.0)	<0.001
Baseline moderate to severe anemia, n (%)	211 (43.1)	39 (10.8)	<0.001
Oral iron therapy, n (%)	324 (66.1)	194 (53.7)	<0.001

Values are expressed as absolute and relative frequencies or as medians and ranges. Benign uterine gynecological conditions include adenomyosis, endometriosis, and uterine fibroids. Gastrointestinal disorders include disorders associated with nutrient malabsorption (e.g., inflammatory bowel disease, celiac disease). Hematologic disorders include thrombocytopenia, Von Willebrand disease, and hemophilia. Respiratory disorders include treated chronic asthma, and uncomplicated pneumonia during pregnancy requiring antibiotic therapy and not requiring respiratory support or intensive care management. Psychiatric disorders include anxiety disorders, mood disorders, and personality disorders requiring pharmacological treatment. Placenta accreta spectrum disorders were defined according to current international classifications. Inadequate prenatal care was defined as initiation of antenatal care after 28 weeks of gestation and/or fewer than four prenatal visits during pregnancy. Infectious diseases during pregnancy, including TORCH infections (Toxoplasmosis, Other infections, Rubella, Cytomegalovirus, and Herpes simplex virus), were recorded. Baseline hemoglobin represented the pre-treatment value for the treated group and the gestational age-matched value in the control group (±2 weeks). Moderate to severe anemia was defined according to baseline hemoglobin values lower than 9.9 g/dL. Oral iron therapy represented the first-line treatment in the treated group, according to international guidelines. BMI: body mass index; GI: gastrointestinal; DM: diabetes mellitus; ART: assisted reproductive technique; GDM: gestational diabetes mellitus; HDPs: hypertensive disorders of pregnancy; PAS: placenta accreta spectrum; Hb: hemoglobin.

**Table 2:** Delivery outcomes with comparisons between the study groups.

Outcome	Treated Group (N = 490)	Control Group (N = 361)	*p*-Value
GA at delivery, days, median (range)	274 (157–294)	274 (168–294)	0.87
Vaginal delivery, n (%)	270 (55.1)	233 (64.5)	<0.01
Cesarean delivery, n (%)	220 (44.9)	128 (35.5)	<0.01
Induction of labor, n (%)	141 (28.8)	132 (36.6)	<0.05
Epidural use, n (%)	134 (27.5)	134 (37.1)	<0.01
PPH, n (%)	110 (22.4)	84 (23.3)	0.76
Blood loss at delivery, ml, median (range)	400 (50–2600)	400 (100–2900)	0.80
Bakri balloon use, n (%)	30 (6.1)	7 (1.9)	<0.01
Postpartum Hb, g/dL, median (range)	9.9 (5.6–13.4)	8.9 (5.4–12.1)	<0.001
Postpartum IV iron therapy, n (%)	59 (12.0)	92 (25.5)	<0.001
Postpartum oral iron therapy, n (%)	264 (54.1)	301 (83.4)	<0.001
Need for blood transfusion, n (%)	19 (3.9)	17 (4.7)	0.55
Birth weight, g, median (range)	3250 (400–4615)	3240 (620–4740)	0.64
Placental weight, g, median (range)	580 (260–1360)	540 (210–1070)	<0.01
Apgar score at 5 min, median (range)	10 (4–10)	10 (0–10)	0.99
Arterial cord blood pH, median (range)	7.27 (6.85–7.50)	7.27 (6.96–7.90)	0.27
NICU or high-dependency care admission, n (%)	93 (19.0)	88 (24.4)	0.05
Perinatal death, n (%)	5 (1.0)	4 (1.1)	0.91
Neonatal jaundice, n (%)	24 (4.9)	31 (8.6)	<0.05

Values are expressed as absolute and relative frequencies or as medians and ranges. Postpartum hemorrhage was defined as blood loss exceeding 500 mL following vaginal delivery and 1000 mL following cesarean delivery. PPH: postpartum hemorrhage; Hb: hemoglobin; IV: intravenous; NICU: neonatal intensive care unit.

**Table 3:** Results from binary logistic regression and generalized linear models for the associations between group, baseline hemoglobin concentrations, and maternal neonatal outcomes.

	Group Effect Estimate		Baseline Hb Effect Estimate	
Outcome	aOR/β (95% CI)	*p*-Value	aOR/β (95% CI)	*p*-Value
Blood transfusion *	0.42 (0.17–0.99)	0.05	0.65 (0.38–1.09)	0.11
Bakri balloon use *	3.38 (0.90–12.5)	0.07	1.38 (0.84–2.27)	0.19
Postpartum IV iron therapy *	0.28 (0.18–0.44)	<0.001	0.68 (0.52–0.88)	<0.01
PPH *	0.80 (0.55–1.16)	0.24	0.84 (0.67–1.06)	0.15
Cesarean delivery **	1.28 (0.91–1.80)	0.14	0.99 (0.81–1.22)	0.98
Birth weight **	20.0 (−77.2; 37.2)	0.49	−30.8 (−65.5; 3.8)	0.08
NICU or high-dependency care admission ***	0.43 (0.26–0.71)	<0.001	1.26 (0.92–1.73)	0.13
Neonatal jaundice *	0.44 (0.21–0.91)	<0.05	1.14 (0.72–1.81)	0.56

* Logistic regression model adjusted for maternal age, pregestational BMI, parity, conception mode, multiple pregnancy, oral iron therapy, baseline Hb value, gestational age at delivery, and delivery mode. ** Logistic regression and generalized linear models adjusted for maternal age, pregestational BMI, parity, conception mode, multiple pregnancy, baseline Hb value, and gestational age at delivery. *** Logistic regression model adjusted for maternal age, pregestational BMI, parity, conception mode, multiple pregnancy, baseline Hb value, gestational age at delivery, and delivery mode.

## Data Availability

The data analyzed in this study were coded/pseudo-anonymized. Due to ethical and institutional policy restrictions, they are available from the corresponding author upon reasonable request.

## Abbreviations

The following abbreviations are used in this manuscript:

ID	Iron Deficiency
Hb	Hemoglobin
FIGO	International Federation of Gynecology and Obstetrics
IDA	Iron Deficiency Anemia
PPH	Postpartum Hemorrhage
FGR	Fetal Growth Restriction
NICU	Neonatal Intensive Care Unit
PBM	Patient Blood Management
IV	Intravenous
RBC	Red Blood Cell
FCM	Ferric Carboxymaltose
GDM	Gestational Diabetes Mellitus
HDPs	Hypertensive Disorders of Pregnancy
PAS	Placenta Accreta Spectrum
BMI	Body Mass Index
aOR	Adjusted Odd Ratio
CI	Confidence Interval
ART	Assisted Reproductive Technique
